# A Descriptive Review of the Antioxidant Effects and Mechanisms of Action of Berberine and Silymarin

**DOI:** 10.3390/molecules29194576

**Published:** 2024-09-26

**Authors:** Ana María García-Muñoz, Desirée Victoria-Montesinos, Pura Ballester, Begoña Cerdá, Pilar Zafrilla

**Affiliations:** Faculty of Pharmacy and Nutrition, UCAM Universidad Católica de Murcia, 30107 Murcia, Spain; amgarcia13@ucam.edu (A.M.G.-M.); dvictoria@ucam.edu (D.V.-M.); bcerda@ucam.edu (B.C.); mpzafrilla@ucam.edu (P.Z.)

**Keywords:** antioxidant activity, *Silybum marianum*, silymarin, berberine, bioactive compounds

## Abstract

Oxidative stress is a key factor in the development of chronic diseases such as type 2 diabetes, cardiovascular diseases, and liver disorders. Antioxidant therapies that target oxidative damage show significant promise in preventing and treating these conditions. Berberine, an alkaloid derived from various plants in the *Berberidaceae* family, enhances cellular defenses against oxidative stress through several mechanisms. It activates the AMP-activated protein kinase (AMPK) pathway, which reduces mitochondrial reactive oxygen species (ROS) production and improves energy metabolism. Furthermore, it boosts the activity of key antioxidant enzymes like superoxide dismutase (SOD), catalase (CAT), and glutathione peroxidase (GPx), thus protecting cells from oxidative damage. These actions make berberine effective in managing diseases like type 2 diabetes, cardiovascular conditions, and neurodegenerative disorders. Silymarin, a flavonolignan complex derived from *Silybum marianum*, is particularly effective for liver protection. It activates the nuclear factor erythroid 2-related factor 2 (Nrf2) pathway, enhancing antioxidant enzyme expression and stabilizing mitochondrial membranes. Additionally, silymarin reduces the formation of ROS by chelating metal ions, and it also diminishes inflammation. This makes it beneficial for conditions like non-alcoholic fatty liver disease (NAFLD) and alcohol-related liver disorders. This review aims to highlight the distinct mechanisms by which berberine and silymarin exert their antioxidant effects.

## 1. Introduction

The growing interest in natural bioactive compounds for health improvement and disease prevention is largely attributed to their potent antioxidant properties. Oxidative stress, caused by an imbalance between reactive oxygen species (ROS) and the body’s antioxidant defenses, plays a crucial role in the pathogenesis of numerous chronic diseases, including cardiovascular diseases, diabetes, and neurodegenerative disorders [1]. Among these natural compounds, berberine and silymarin have been widely studied for their potent antioxidant activities and mechanisms of action.

Berberine, an isoquinoline alkaloid, is derived from several medicinal plants, including *Berberis aristata* and *Coptis chinensis* [2]. Traditionally used in Chinese and Ayurvedic medicine, berberine demonstrates significant antioxidant activity through multiple mechanisms [3]. Primarily, it enhances the activity of endogenous antioxidant enzymes such as superoxide dismutase (SOD), glutathione peroxidase (GPx), and catalase (CAT), which are critical in neutralizing ROS and preventing cellular damage [4]. Berberine activates the AMP-activated protein kinase (AMPK) pathway, which not only regulates energy homeostasis but also inhibits mitochondrial ROS production, thus protecting cells from oxidative damage [5]. Additionally, berberine exhibits direct free radical scavenging activity, further contributing to its role as a powerful antioxidant [6]. Structurally, berberine is an isoquinoline alkaloid characterized by a planar, tetracyclic framework with a quaternary ammonium group. This rigid, aromatic structure is critical for its biological activity, allowing berberine to intercalate with DNA and interact with enzymes involved in oxidative stress modulation [7].

Silymarin, a flavonolignan complex extracted from the seeds of *Silybum marianum* (milk thistle), is renowned for its hepatoprotective effects, but its antioxidant properties are equally significant [8]. Silymarin’s antioxidant mechanism involves the inhibition of ROS-producing enzymes, stabilization of mitochondrial membranes under oxidative stress, and activation of the nuclear factor erythroid 2-related factor 2 (Nrf2) pathway [9]. This pathway enhances the expression of various antioxidant enzymes, improving the cellular redox balance [5,10]. Furthermore, silymarin’s ability to chelate metal ions prevents the formation of highly reactive hydroxyl radicals, a key contributor to oxidative stress [11]. Silymarin’s structure is more complex than that of berberine, as it is composed of a mixture of flavonolignans, primarily silibinin, along with silychristin and silydianin. Silibinin, the main active component, consists of two flavonoid units linked to lignan structures [12].

Berberine and silymarin exhibit significant antioxidant activity by enhancing endogenous defenses and scavenging free radicals, leading to a reduction in oxidative stress. This effect is particularly relevant in managing metabolic and inflammatory conditions such as diabetes, cardiovascular diseases, and neurodegenerative disorders [5,13]. Additionally, their anti-inflammatory and metabolic regulatory properties further contribute to their therapeutic potential, making them valuable for addressing a broad spectrum of health conditions [11,14].

Despite their potent biological effects, both berberine and silymarin face challenges related to poor bioavailability, which limits their clinical efficacy. Berberine demonstrates low oral absorption due to its poor solubility and rapid first-pass metabolism, resulting in low plasma concentrations [15]. To address this, strategies such as nanoparticle-based delivery systems (e.g., liposomes and phytosomes) [16] and combining berberine with piperine to inhibit metabolic degradation have been proposed [17].

Similarly, silymarin’s bioavailability is restricted by its poor solubility and rapid metabolic clearance [18]. Formulations like silymarin phytosomes have been developed to improve its absorption and therapeutic efficacy in managing oxidative stress and metabolic diseases [18].

Both compounds’ limited bioavailability has been a significant hurdle in clinical application, but these advancements in formulation technology are paving the way for more effective use of berberine and silymarin in therapeutic settings.

To develop this review, we conducted a thorough search using PubMed, Scopus, and Web of Science databases. Keywords such as “berberine”, “silymarin”, “antioxidant”, “oxidative stress”, and “bioavailability” were used. Inclusion criteria focused on peer-reviewed studies with high methodological quality, including well-defined experimental designs and robust data analysis, particularly those offering detailed insights into the antioxidant mechanisms of berberine and silymarin.

This review aims to consolidate and present a comprehensive overview of the antioxidant capacities of berberine and silymarin, focusing on how their bioactive compounds and mechanisms of action contribute to their potential in managing oxidative stress-related conditions.

## 2. Antioxidant Activity of Berberine and Silymarin: Mechanism of Action

### 2.1. Antioxidant Activity of Berberine

#### 2.1.1. Overview

Berberine exhibits significant antioxidant properties through multiple biochemical pathways and mechanisms. These mechanisms collectively contribute to berberine’s efficacy in neutralizing oxidative stress and protecting cellular components from damage [19,20].

#### 2.1.2. Enhancement of Endogenous Antioxidant Enzymes

Primarily, berberine enhances the activity of endogenous antioxidant enzymes, which play a crucial role in mitigating oxidative stress [21,22,23]. This enhancement occurs through several mechanisms, including the upregulation of gene expression and the stabilization of these enzymes’ structures [21]. Studies have shown that berberine increases the activity of SOD, CAT, and GPx. SOD catalyzes the dismutation of superoxide radicals into hydrogen peroxide and oxygen, which is a critical first step in mitigating oxidative stress, as superoxide radicals are highly reactive and can cause significant cellular damage [24]. The subsequent action of CAT, which converts hydrogen peroxide into water and oxygen, is vital because hydrogen peroxide can diffuse through membranes and potentially generate hydroxyl radicals via Fenton reactions if it is not rapidly neutralized [25]. GPx reduces lipid hydroperoxides to their corresponding alcohols and free hydrogen peroxide to water, thus preventing lipid peroxidation, which can compromise cell membrane integrity and function [26]. This enhancement of antioxidant enzyme activity by berberine is vital in maintaining cellular redox balance and protecting against oxidative damage by ensuring that reactive oxygen species are effectively neutralized [22,23].

It also seems that berberine induces the expression of CAT and SOD isozymes, which significantly contribute to its antioxidant activity [27]. This induction increases the total activity of these enzymes, thereby enhancing the cell’s capacity to neutralize ROS and protect against oxidative damage. The specific mechanism by which berberine enhances the expression of CAT and SOD involves several pathways and regulatory mechanisms [27].

Berberine’s influence on the expression and activity of these enzymes has been demonstrated in various studies. For instance, total CAT activity was shown to increase approximately 2.1-fold upon exposure to berberine, with the increase attributed to the induction of the CAT2 isozyme [23]. Similarly, total SOD activity increased approximately 1.7-fold, with the SOD2 and SOD3 isozymes contributing to this enhancement [23]. This increase in enzymatic activity is crucial for the detoxification of superoxide radicals and hydrogen peroxide, thereby reducing oxidative stress and protecting cellular components from oxidative damage [23].

The upregulation of these enzymes is particularly important in conditions where oxidative stress is elevated, such as in inflammatory diseases and metabolic disorders [28]. In these pathological states, the production of ROS exceeds the neutralizing capacity of endogenous antioxidants, leading to cellular damage and dysfunction. By increasing the levels of CAT and SOD, berberine enhances the antioxidant defense system, thereby mitigating the harmful effects of excessive ROS [14]. This upregulation not only helps in neutralizing ROS but also in maintaining cellular redox homeostasis, which is essential for normal cellular functions and survival [23].

The mechanism behind berberine-induced upregulation of CAT and SOD involves its interaction with various signaling pathways. Berberine activates the Nrf2 pathway, which plays a central role in the regulation of antioxidant genes [29]. Upon activation, Nrf2 translocates to the nucleus and binds to AREs in the promoters of target genes, including those encoding for CAT and SOD [30]. This binding enhances the transcription of these genes, leading to increased synthesis and activity of the respective enzymes [23]. Additionally, berberine’s activation of the AMPK pathway contributes to the upregulation of these antioxidant enzymes. AMPK activation has been shown to enhance the expression of antioxidant enzymes through various mechanisms, including the stabilization of Nrf2 and the enhancement of its nuclear translocation [24]. By activating both Nrf2 and AMPK pathways, berberine ensures a robust increase in the levels of CAT and SOD, providing comprehensive protection against oxidative stress [23].

#### 2.1.3. Direct Free Radical Scavenging Activity

In addition to augmenting endogenous antioxidant enzymes, berberine exhibits direct free radical scavenging activity [23]. This property has been demonstrated through various in vitro assays, including the DPPH (2,2-diphenyl-1-picrylhydrazyl) and ABTS (2,2′-azino-bis (3-ethylbenzothiazoline-6-sulphonic acid)) radical scavenging assays [31]. These assays are commonly used to measure the antioxidant capacity of compounds due to their sensitivity and reproducibility. In these assays, berberine shows a capacity to neutralize free radicals in a dose-dependent manner, effectively reducing oxidative stress within the cellular environment [32]. The free radical scavenging activity of berberine is primarily due to its ability to donate electrons or hydrogen atoms [33]. By donating an electron or hydrogen atom, berberine stabilizes reactive species, converting them into less reactive or non-reactive forms [33]. This action prevents the reactive species from initiating chain reactions that can lead to extensive cellular damage, including lipid peroxidation, protein oxidation, and DNA strand breaks [34]. Thus, berberine’s direct scavenging of free radicals is an important mechanism that complements its role in enhancing endogenous antioxidant defenses, providing a multifaceted approach to reducing oxidative stress [14].

#### 2.1.4. Metal Ion Chelation

Berberine’s antioxidant effect is further amplified through its ability to chelate transition metal ions such as iron and copper [23]. These metal ions catalyze the production of highly reactive hydroxyl radicals via Fenton and Haber–Weiss reactions [35]. In these reactions, ferrous iron (Fe^2+^) or cuprous copper (Cu^+^) reduce hydrogen peroxide (H_2_O_2_) to produce hydroxyl radicals (·OH) and hydroxide ions (OH^−^) [36]. Hydroxyl radicals are among the most reactive and damaging ROS, capable of causing significant oxidative damage to cellular components such as lipids, proteins, and nucleic acids [23,37]. Berberine’s chelation of these metal ions involves the formation of stable complexes with iron and copper, effectively sequestering these metals and preventing them from participating in Fenton reactions. The chelation occurs through berberine’s hydroxyl and methoxy groups, which can donate electron pairs to form coordinate bonds with metal ions. This binding alters the redox potential of the metal ions, rendering them less reactive and thereby inhibiting their ability to catalyze the formation of hydroxyl radicals [22,38]. The significance of this chelation mechanism lies in its ability to reduce metal-induced oxidative stress [39]. In pathological conditions where there is an accumulation of free iron or copper, such as in certain neurodegenerative diseases (e.g., Alzheimer’s and Parkinson’s diseases), cardiovascular diseases, and chronic inflammatory conditions, the Fenton reaction can lead to excessive oxidative damage [39]. By chelating these transition metals, berberine not only prevents the formation of hydroxyl radicals but also helps maintain cellular homeostasis by reducing the overall oxidative burden [22,23]. Moreover, the chelation of metal ions by berberine can also protect cellular structures, particularly the lipid membranes, from peroxidation [40]. Lipid peroxidation is a chain reaction initiated by ROS that results in the degradation of lipids, compromising membrane integrity and function [40]. By preventing the formation of hydroxyl radicals, berberine helps to preserve membrane structure and function, thus protecting cells from oxidative damage and maintaining their viability and normal physiological functions [39,41].

#### 2.1.5. Activation of the Nrf2 Pathway

A key pathway in berberine’s antioxidant action is the Nrf2 pathway [42]. When activated, Nrf2 translocates to the nucleus, where it binds to antioxidant response elements (AREs) in the DNA [42]. This interaction stimulates the transcription of several antioxidant and cytoprotective genes, such as those encoding for SOD, CAT, GPx, and heme oxygenase-1 (HO-1) [21]. Berberine has been shown to enhance the nuclear accumulation of Nrf2, thereby significantly strengthening the cellular antioxidant defense systems [43]. The activation of Nrf2 leads to the expression of genes that increase cellular capacity to neutralize oxidative stress, thus protecting against cellular damage [43]. This enhancement occurs through several pathways, including the PI3K/Akt pathway [43]. Activation of the PI3K/Akt pathway promotes the phosphorylation and subsequent nuclear translocation of Nrf2, which then activates the transcription of ARE-driven genes [43]. The Nrf2 pathway is essential for coordinating the cellular response to oxidative stress [43]. Under normal conditions, Nrf2 is sequestered in the cytoplasm by Kelch-like ECH-associated protein 1 (Keap1), which promotes its ubiquitination and subsequent degradation [44]. During oxidative stress, alterations in the cysteine residues of Keap1 result in the release of Nrf2, enabling its translocation to the nucleus [44]. Berberine has been found to disrupt the Nrf2–Keap1 interaction, allowing Nrf2 to migrate to the nucleus and activate the antioxidant response [45]. Notably, berberine induces the expression of heme oxygenase-1 (HO-1) via the Nrf2 pathway [46]. HO-1 plays a crucial role in breaking down heme into biliverdin, free iron, and carbon monoxide [47], with biliverdin and its metabolite bilirubin acting as potent antioxidants, while carbon monoxide exerts anti-inflammatory effects [47]. Berberine-induced upregulation of HO-1 not only provides direct antioxidant protection but also modulates the cellular redox state and mitigates inflammation [48]. Additionally, berberine’s activation of the Nrf2 pathway has been linked to its neuroprotective effects [14]. Studies have demonstrated that berberine protects against oxidative damage in neuronal cells by enhancing the expression of Nrf2-regulated antioxidant genes [14]. This is particularly relevant in the context of neurodegenerative diseases, where oxidative stress plays a crucial role in pathogenesis [14]. Furthermore, the interplay between the Nrf2 and AMPK pathways contributes to the antioxidant activity of berberine [24]. AMPK activation leads to increased Nrf2 activity and enhances the expression of Nrf2-target genes, further boosting the antioxidant defenses of the cell [49]. Berberine has been shown to activate the AMPK pathway, which in turn promotes the activation of Nrf2, creating a positive feedback loop that amplifies the antioxidant response [50].

#### 2.1.6. Activation of the AMPK Pathway

As discussed earlier, berberine stimulates the AMPK pathway, a key regulator of cellular energy homeostasis and oxidative stress [51,52]. AMPK functions as an energy sensor, balancing cellular energy by promoting ATP-generating catabolic pathways and suppressing ATP-consuming anabolic pathways [53]. Berberine’s activation of AMPK results in the inhibition of NADPH oxidase, an enzyme complex involved in the production of ROS [54]. NADPH oxidase facilitates the transfer of electrons from NADPH to oxygen, producing superoxide anions, which can subsequently form other ROS. By reducing the activity of NADPH oxidase, berberine lowers ROS levels in cells, thereby offering protection against oxidative damage [55].

Furthermore, AMPK activation enhances the expression of uncoupling protein 2 (UCP2), a mitochondrial protein that reduces mitochondrial ROS production by dissipating the proton gradient across the mitochondrial membrane [56]. This reduction in mitochondrial ROS production further contributes to the overall decrease in oxidative stress within the cell [56]. Berberine’s impact on the AMPK pathway also includes the upregulation of antioxidant enzymes [24]. Studies have shown that AMPK activation is linked to increased expression of SOD and other antioxidant enzymes, enhancing the cell’s capacity to neutralize ROS and protect against oxidative damage [57,58].

The regulation of the AMPK pathway by berberine highlights its role in energy metabolism and its indirect antioxidant effects through modulation of cellular ROS production pathways [14]. Studies have shown that AMPK activation is linked to increased expression of SOD and other antioxidant enzymes, enhancing the cell’s capacity to neutralize ROS and protect against oxidative damage [23,59].

### 2.2. Antioxidant Activity of Silymarin

#### 2.2.1. Overview

Silymarin is recognized for its potent antioxidant properties [60]. The primary constituents of silymarin, including silybin, silydianin, and silychristin, each contribute to its multifaceted antioxidant mechanisms [61]. These mechanisms include direct scavenging of ROS, inhibition of ROS-producing enzymes, maintenance of mitochondrial integrity, activation of antioxidant enzymes and transcription factors, and modulation of cellular redox homeostasis [60].

#### 2.2.2. Free Radical Scavenging and Enzyme Inhibition

Silymarin’s antioxidant defenses operate through multiple pathways. Firstly, it acts as a direct scavenger of free radicals. The phenolic hydroxyl groups present in silymarin’s structure enable it to donate hydrogen atoms to free radicals, thereby neutralizing them and preventing oxidative damage [62]. Studies have shown that silybin, a major component of silymarin, is effective in scavenging hypochlorous acid (HOCl) and hydroxyl radicals, although it is less effective against superoxide anions [63]. This direct scavenging activity helps reduce the overall oxidative burden within cells [63].

To further elaborate on the free radical scavenging mechanism, silybin’s structure allows it to interact efficiently with various ROS. The effectiveness of silybin in scavenging HOCl is particularly noteworthy, as HOCl is a potent oxidant produced by neutrophils during inflammatory responses [64,65]. By neutralizing HOCl, silymarin can mitigate oxidative damage and inflammation simultaneously [64]. Additionally, silybin’s ability to scavenge hydroxyl radicals, which are among the most reactive and damaging ROS, underscores its importance in cellular protection [66]. Hydroxyl radicals can initiate lipid peroxidation, protein oxidation, and DNA strand breaks, leading to significant cellular dysfunction. The phenolic hydroxyl groups in silybin donate hydrogen atoms to these radicals, forming more stable and less reactive species, thus terminating the chain reactions that cause extensive cellular damage [64].

Despite its lesser efficacy against superoxide anions, silymarin’s overall antioxidant potential is amplified through its combined actions against multiple ROS [64]. The differential scavenging capacities of silymarin’s constituents, such as silydianin and silychristin, which have been reported to be 2–10 times more active than silybin in certain conditions, contribute to a broad-spectrum antioxidant defense [62]. This diversity in activity among the components ensures a comprehensive approach to neutralizing a wide range of oxidative threats [64].

Moreover, the diffusion-controlled rate constants of silybin in relation to hydroxyl radicals suggest that it can rapidly neutralize these radicals in high-stress environments [64]. This rapid response is crucial in preventing the initial stages of oxidative damage, which can escalate quickly if not promptly addressed. Silybin’s ability to decrease H_2_O_2_ levels in stressed neuronal cells further highlights its protective role in oxidative stress conditions [62].

In addition to its direct scavenging capabilities, silymarin prevents the formation of free radicals by inhibiting specific enzymes responsible for ROS production and by maintaining the integrity of the mitochondrial electron transport chain under stress conditions [62]. Mitochondria are the primary sites of ROS production during oxidative phosphorylation. Silymarin supports mitochondrial function by optimizing the electron transport chain, reducing electron leakage, and directly inhibiting ROS-producing enzymes within mitochondria [62]. This protection helps to maintain ATP levels, membrane potential, and overall mitochondrial bioenergetics, thereby reducing oxidative stress.

Silymarin enhances the efficiency of the electron transport chain (ETC), specifically by stabilizing the complexes involved in electron transfer, which reduces electron leakage that can form superoxide anions. By minimizing these leakages, silymarin helps in maintaining the efficiency of oxidative phosphorylation and ATP production [64].

Furthermore, silymarin has been shown to inhibit the activity of enzymes such as NADPH oxidase and xanthine oxidase, which are significant sources of ROS. NADPH oxidase catalyzes the transfer of electrons from NADPH to oxygen, forming superoxide anions [11]. By inhibiting NADPH oxidase, silymarin effectively reduces the formation of these harmful radicals. Similarly, xanthine oxidase, which is involved in purine metabolism, can produce superoxide anions during its activity. Silymarin’s inhibitory effect on xanthine oxidase further decreases ROS production, contributing to a lower oxidative stress environment within the cell [11].

The role of silymarin in preserving mitochondrial function is further emphasized by its ability to protect against mitochondrial dysfunction during ischemia/reperfusion injury [67]. Studies have shown that silymarin can prevent significant impairments in mitochondrial bioenergetics, such as decreased ATP levels, membrane potential, and respiration rates, by stabilizing mitochondrial membranes and reducing ROS formation [62,68]. This protective effect is vital in conditions where oxidative stress is exacerbated by compromised mitochondrial function [64]. Additionally, silymarin has been observed to enhance the expression of mitochondrial protective proteins. By upregulating the expression of these proteins, silymarin aids in maintaining mitochondrial integrity and function under oxidative stress conditions. This action ensures that cells can sustain their energy requirements and reduce the likelihood of apoptosis induced by mitochondrial dysfunction [64].

#### 2.2.3. Activation of Antioxidant Enzymes and Transcription Factors

Silymarin also enhances the activity of endogenous antioxidant enzymes and non-enzymatic antioxidants. This enhancement is primarily mediated through the activation of transcription factors such as Nrf2 and nuclear factor-kappa B (NF-κB) [62]. Activation of Nrf2 by silymarin leads to its translocation to the nucleus, where it binds to AREs in the DNA, promoting the transcription of genes encoding for antioxidant enzymes such as SOD, CAT, GPx, and HO-1 [62]. As mentioned in Section 2.1.5, HO-1 plays a crucial role in degrading heme into biliverdin, iron, and carbon monoxide, with biliverdin and bilirubin (its metabolite) acting as potent antioxidants [47].

Therefore, silymarin enhances the antioxidant response by facilitating the activation of Nrf2, leading to the upregulation of cytoprotective proteins [69]. This process bolsters the cell’s defense mechanisms against oxidative damage, further emphasizing its role in mitigating oxidative stress [9]. Additionally, Nrf2 activation by silymarin contributes to the upregulation of phase II detoxifying enzymes, further enhancing the cell’s ability to neutralize harmful substances and mitigate oxidative stress [70].

Similarly, silymarin’s impact on NF-κB, a transcription factor that plays a pivotal role in inflammation and immune responses, further augments its antioxidant effects [62]. NF-κB is normally held inactive in the cytoplasm by IκB proteins [71]. Upon activation by oxidative stress or pro-inflammatory signals, IκB is degraded, allowing NF-κB to translocate to the nucleus and initiate the transcription of genes involved in inflammation and cell survival [71]. Modulating NF-κB activity, silymarin not only enhances antioxidant defenses but also reduces inflammation, which is closely linked with oxidative stress [64]. This dual action helps to maintain cellular homeostasis under stress conditions. Moreover, the inhibition of NF-κB by silymarin reduces the expression of pro-inflammatory cytokines and adhesion molecules, thereby diminishing inflammatory responses and contributing to improved cellular resilience [72].

#### 2.2.4. Regulation of Stress Response Genes

Silymarin also contributes to cellular redox homeostasis by activating vitagenes, which are involved in the synthesis of protective molecules [62]. These include heat shock proteins (HSPs), thioredoxin (Trx), and sirtuins, which play essential roles in maintaining cellular function and integrity under stress conditions [73]. Vitagenes are a group of genes that become activated in response to stress and help to maintain cellular homeostasis [73]. By upregulating these vitagenes, silymarin enhances the cell’s ability to cope with oxidative stress and maintain optimal redox balance [74].

HSPs function as molecular chaperones, ensuring proper protein folding and preventing aggregation of misfolded proteins [75]. Their upregulation by silymarin aids in protecting cells against damage induced by oxidative stress [62]. HSPs help to stabilize proteins and cellular structures, ensuring that they maintain their function even under adverse conditions. This is particularly important during oxidative stress, where the risk of protein denaturation and aggregation increases significantly [64]. HSPs also interact with the cellular machinery responsible for protein degradation, facilitating the removal of damaged proteins and thus preventing the accumulation of toxic protein aggregates [75]. This dual role of HSPs in both protein folding and degradation underscores their importance in maintaining protein homeostasis and cellular integrity under stress conditions [76].

Trx is another critical component upregulated by silymarin that helps to reduce oxidized proteins and maintain the redox state within cells [77]. Trx is involved in disulfide bond reduction, which is crucial for maintaining the structure and function of many proteins [73]. By keeping proteins in their reduced and functional state, Trx helps to prevent oxidative damage and ensures the proper functioning of various cellular processes [73]. Additionally, Trx plays a role in regulating the activity of transcription factors and enzymes involved in the cellular stress response, thereby modulating the expression of genes that are essential for antioxidant defense and repair mechanisms [73].

Sirtuins, a family of NAD+-dependent deacetylases, play significant roles in extending cellular lifespan, enhancing stress resistance, and improving metabolic efficiency [73]. Sirtuins are involved in regulating mitochondrial biogenesis, DNA repair, and apoptosis, all of which are essential for maintaining cellular health under stress conditions [73]. By enhancing the expression of sirtuins, silymarin helps to bolster the cell’s defenses against oxidative damage and improve overall cellular resilience [64]. Sirtuins also influence metabolic pathways by modulating the activity of enzymes involved in glucose and lipid metabolism, thus contributing to improved energy homeostasis and reduced accumulation of metabolic by-products that can exacerbate oxidative stress [73].

**Figure 1:**
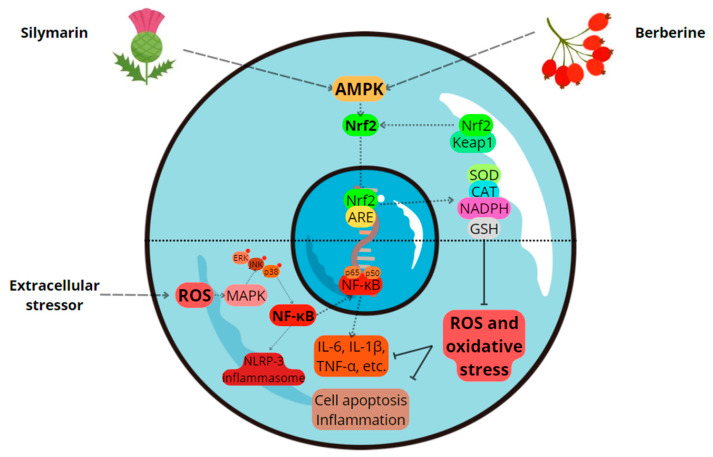
Mechanisms of Antioxidant Action of Berberine and Silymarin.

By enhancing the expression of these protective molecules, silymarin ensures robust cellular defense mechanisms against oxidative and other forms of stress. This multi-faceted approach not only improves the cell’s ability to handle oxidative stress but also enhances overall cellular function and longevity. The activation of vitagenes by silymarin represents a critical aspect of its antioxidant activity, providing a broad-spectrum defense against various stressors and contributing to the maintenance of cellular homeostasis and optimal redox balance [64]. Table 1 and Figure 1 summarize the antioxidant activity and mechanisms of action of berberine and silymarin.

## 3. Anti-Inflammatory Activity of Berberine and Silymarin

### 3.1. Anti-Inflammatory Activity of Berberine

#### 3.1.1. Inhibition of NF-κB and AP-1 Pathways

Berberine has been shown to exert potent anti-inflammatory effects by targeting and inhibiting the NF-κB [64] (nuclear factor kappa-light-chain-enhancer of activated B cells) and AP-1 (activator protein 1) signaling pathways [78]. Both transcription factors are crucial in regulating pro-inflammatory genes and play essential roles in the body’s inflammatory response.

NF-κB is activated by various stimuli, such as cytokines, stress, free radicals, and bacterial or viral antigens [71]. Upon activation, NF-κB moves to the nucleus, where it binds to specific DNA sequences, promoting the transcription of inflammation-related genes, including TNF-α (tumor necrosis factor-alpha), IL-1β (interleukin-1 beta), IL-6 (interleukin-6), and MCP-1 (monocyte chemoattractant protein-1) [71]. These cytokines and chemokines are key mediators of inflammation, helping to recruit and activate immune cells at inflammatory sites. Berberine inhibits NF-κB activation by preventing IκB degradation [64].

Similarly, AP-1 regulates the expression of inflammatory genes [78]. It consists of proteins from the JUN (Jun proto-oncogene), FOS (Fos proto-oncogene), ATF (activating transcription factor), and MAF (musculoaponeurotic fibrosarcoma) families, which are activated by signals such as cytokines, growth factors, and stress [78]. These transcription factors play key roles in cell proliferation, differentiation, and apoptosis, contributing to the regulation of inflammatory responses. Once activated, AP-1 translocates to the nucleus, binding to TPA-responsive elements (TREs) in DNA to promote the transcription of genes involved in inflammation, cell proliferation, and differentiation [78]. Berberine blocks AP-1 activity by preventing its components from binding to DNA [79], which reduces the expression of key enzymes such as COX-2 (cyclooxygenase-2) and iNOS (inducible nitric oxide synthase). COX-2 is involved in prostaglandin production, mediating inflammation and pain, while iNOS generates nitric oxide, contributing to tissue damage and inflammation. By inhibiting AP-1, berberine decreases the production of these enzymes, thus mitigating inflammation [79].

The simultaneous inhibition of NF-κB and AP-1 by berberine results in a robust suppression of inflammatory responses [64,79]. Both transcription factors regulate a wide range of pro-inflammatory cytokines, chemokines, and enzymes. By targeting these pathways, berberine effectively reduces the production of TNF-α, IL-1β, IL-6, MCP-1, COX-2, and iNOS, leading to a substantial decrease in inflammation and its symptoms [79].

#### 3.1.2. Activation of AMP-Activated Protein Kinase

Berberine activates AMPK, a key regulator of cellular energy balance and inflammation, as discussed in Section 2.1.6. By activating AMPK, berberine plays a significant role in reducing the expression of pro-inflammatory genes [51,52].

In macrophages, this activation of AMPK by berberine leads to a notable reduction in several key inflammatory markers, including TNF-α, IL-1β, IL-6, MCP-1, iNOS, and COX-2 [79]. These mediators are crucial for driving inflammation, with TNF-α and IL-1β being primary cytokines that initiate inflammatory processes, IL-6 being involved in the acute phase response, and MCP-1 playing a role in monocyte recruitment to inflammation sites [79,80].

Berberine activates AMPK through multiple mechanisms, with one primary mechanism being the inhibition of mitochondrial respiratory complex I, which increases the AMP/ATP ratio. This rise activates AMPK, leading to the phosphorylation and inhibition of acetyl-CoA carboxylase (ACC), an enzyme essential for fatty acid synthesis. Inhibiting ACC lowers malonyl-CoA levels, reducing lipid synthesis while promoting fatty acid oxidation [81,82].

Further studies have shown that berberine’s anti-inflammatory effects via AMPK activation are negated when AMPK inhibitors or dominant-negative AMPK are used [79], underscoring the importance of AMPK activation in this process. AMPK activation also inhibits the mTOR pathway, which regulates cell growth and inflammation. By suppressing mTOR, berberine further reduces the expression of pro-inflammatory cytokines [79].

#### 3.1.3. Modulation of Gut Microbiota and Treg/Th17 Balance

Berberine’s anti-inflammatory effects also extend to influencing the gut microbiota and the balance between regulatory T cells (Treg) and T-helper 17 cells (Th17), which are vital for immune homeostasis and controlling inflammation [83].

The gut microbiota consists of a diverse community of microorganisms that play a critical role in regulating immune responses and overall health [84]. Disruptions in gut microbiota composition, known as dysbiosis, have been associated with various inflammatory and autoimmune disorders [84]. Berberine has been shown to positively modulate gut microbiota composition [85], increasing beneficial bacteria such as *Akkermansia*, *Oscillibacter*, *Lachnospiraceae*, and *Ruminococcaceae* while decreasing potentially harmful bacteria like *Lactobacilli* [85].

*Akkermansia* strengthens the intestinal barrier and has anti-inflammatory effects by suppressing IFNγ, leading to a reduced Th17 response. *Lachnospiraceae* and *Ruminococcaceae* are butyrate-producing bacteria [85]. Butyrate, a short-chain fatty acid, plays a key role in promoting Treg cell development and inhibiting Th17 differentiation via G-protein-coupled receptor signaling and histone deacetylase inhibition [86]. Berberine’s ability to modulate gut microbiota helps maintain the Treg/Th17 balance, which is critical in preventing excessive inflammatory responses [86].

Berberine also promotes the differentiation of naive CD4+ T cells into Tregs rather than Th17 cells [86]. This is achieved through the activation of the aryl hydrocarbon receptor (AhR) and the upregulation of cytochrome P450 1A1 (CYP1A1) [87]. AhR is a transcription factor that induces the expression of CYP1A1, which in turn enhances Foxp3 expression, a key transcription factor for Treg cell function [87].

Foxp3+ Treg cells are crucial for maintaining immune tolerance and preventing excessive inflammatory responses. They achieve this by secreting anti-inflammatory cytokines such as IL-10, IL-35, and TGF-β, which suppress the differentiation and function of pro-inflammatory Th1 and Th17 cells [83]. By promoting the differentiation and function of Tregs, berberine helps to limit autoimmune and inflammatory responses, contributing to its anti-inflammatory properties.

Th17 cells are pro-inflammatory cells that produce cytokines such as IL-17, IL-21, and IL-22, which are implicated in autoimmune and inflammatory diseases. The differentiation of Th17 cells is driven by cytokines like TGF-β, IL-6, and IL-23, and involves the activation of transcription factors such as RORγt and STAT3 [88]. Berberine has been shown to inhibit Th17 cell differentiation by downregulating these cytokines and transcription factors [86].

Additionally, berberine’s modulation of gut microbiota indirectly impacts Th17 cell differentiation. The increased production of butyrate and other short-chain fatty acids by beneficial gut bacteria, promoted by berberine, inhibits Th17 cell differentiation and supports Treg development. This shift from a pro-inflammatory Th17 response to an anti-inflammatory Treg response is critical for reducing inflammation and autoimmunity [83].

#### 3.1.4. Inhibition of Mitogen-Activated Protein Kinase (MAPK) Pathways

Berberine inhibits the mitogen-activated protein kinase (MAPK) signaling pathways, which play a crucial role in the production of pro-inflammatory cytokines. By suppressing these pathways, berberine reduces reactive oxygen species (ROS) production and inflammatory cytokine levels, thus contributing to inflammation reduction in various tissues [79].

The MAPK pathways are essential signaling cascades that control cellular responses to external stimuli like stress, cytokines, and growth factors. These pathways include extracellular signal-regulated kinase (ERK), c-Jun N-terminal kinase (JNK), and p38 MAPK pathways [89]. Each of these pathways is activated by distinct stimuli, leading to the activation of specific transcription factors that regulate genes involved in inflammation, apoptosis, and cell differentiation [89].

Berberine’s inhibition of MAPK pathways occurs through various mechanisms. It has been shown to suppress ERK1/2 activation in different cell types, including macrophages and hepatocytes. The ERK1/2 pathway, activated by mitogens, is involved in cell proliferation and differentiation. By inhibiting ERK1/2 activation, berberine lowers the transcription of pro-inflammatory genes and the production of cytokines such as TNF-α and IL-6 [79].

In addition, berberine inhibits the JNK pathway, which is typically triggered by stress signals such as UV radiation and inflammatory cytokines. JNK activation leads to the phosphorylation of transcription factors like c-Jun, which drives the expression of pro-inflammatory genes. By blocking the JNK pathway, berberine reduces c-Jun activation and subsequently decreases the production of inflammatory cytokines, thus alleviating inflammation [79].

Berberine also suppresses p38 MAPK activation, which is induced by environmental stresses and inflammatory cytokines. p38 MAPK plays a vital role in the production of pro-inflammatory cytokines and the initiation of inflammatory responses. By inhibiting p38 MAPK, berberine reduces the levels of pro-inflammatory mediators such as IL-1β and TNF-α, which is especially important in mitigating chronic inflammation and tissue damage [79,82].

Since the MAPK pathways regulate ROS production, berberine’s inhibition of these pathways leads to a reduction in ROS levels, which helps to decrease oxidative stress and the inflammation it causes [79,82].

The anti-inflammatory effects of berberine via MAPK pathway inhibition are supported by in vivo studies. For example, animal models of inflammation treated with berberine showed decreased levels of phosphorylated ERK, JNK, and p38 MAPK, correlating with reduced inflammatory cytokine production and improved inflammatory outcomes. These findings underscore the therapeutic potential of berberine in managing inflammation through the modulation of MAPK signaling [79,82].

#### 3.1.5. Inhibition of Pro-Inflammatory Cytokine Production

Berberine exerts significant effects on macrophages and other immune cells, promoting a shift from the pro-inflammatory M1 phenotype to the anti-inflammatory M2 phenotype. This shift results in decreased production of pro-inflammatory cytokines and increased release of anti-inflammatory mediators such as IL-10. This modulation of immune cell activity is crucial for reducing the overall inflammatory response [83].

Berberine also reduces endotoxin-induced cytokine release, a critical aspect in conditions involving bacterial infections and sepsis. By inhibiting signaling pathways activated by endotoxins like lipopolysaccharides (LPSs), berberine decreases the release of TNF-α, IL-6, and IL-1β from immune cells, thereby dampening the exaggerated inflammatory response typically seen in these conditions [81].

Furthermore, berberine inhibits the activation of inflammasomes, particularly the NLRP3 inflammasome, which is involved in the maturation and release of IL-1β and IL-18. Inhibition of inflammasome activation by berberine reduces the production of these potent pro-inflammatory cytokines, adding another layer of its anti-inflammatory action [24,82].

### 3.2. Anti-Inflammatory Activity of Silymarin

Silymarin exhibits significant anti-inflammatory properties through various mechanisms, which collectively contribute to its ability to reduce the production of pro-inflammatory cytokines. Here, we explore the key mechanisms by which silymarin achieves these effects, focusing on its modulation of cytokine signaling pathways, its impact on immune cells, reduction of endotoxin-induced cytokine release, and inhibition of inflammasome activation.

#### 3.2.1. Modulation of Cytokine Signaling Pathways

Silymarin, particularly through its active component silibinin, exerts its anti-inflammatory effects by modulating cytokine signaling pathways, primarily through the inhibition of the NF-κB pathway [90].

Silymarin also downregulates the TLR4/NF-κB-mediated signaling pathways. Toll-like receptor 4 (TLR4) is known to activate NF-κB in response to bacterial LPSs, leading to the production of pro-inflammatory cytokines [90]. By downregulating this pathway, silymarin further decreases the expression of inflammatory mediators such as TNF-α, IL-1β, IL-6, IL-12, IL-23, CCL4, and CXCL10. This broad-spectrum inhibition underscores the potent anti-inflammatory effects of silymarin in various inflammatory conditions [90].

Moreover, silymarin modulates the activity of other signaling molecules involved in the LPS-induced response. It inhibits the activation of MAPK pathways, including ERK1/2 and p38 MAPK, which are also activated by LPSs and contribute to the production of inflammatory mediators. By inhibiting these pathways, silymarin further reduces the expression of pro-inflammatory cytokines and mediators [91].

Studies have demonstrated that silymarin can significantly decrease the levels of several key pro-inflammatory cytokines in LPS-stimulated cells. For example, in murine macrophage cell lines (RAW 264.7 cells), silymarin treatment reduced the production of TNF-α, IL-1β, and IL-6 following LPS exposure. These effects were associated with a decrease in the activation of NF-κB and MAPK signaling pathways, indicating that silymarin’s anti-inflammatory actions were mediated through these pathways [90].

Furthermore, silymarin has been shown to reduce the production of nitric oxide (NO) in response to LPSs. Nitric oxide is produced by iNOS during inflammatory responses and contributes to inflammation and tissue damage. Silymarin inhibits the expression of iNOS by blocking NF-κB activation, thus decreasing NO production and alleviating inflammation [90].

Silymarin also impacts other signaling pathways involved in inflammation. For instance, it inhibits the activation of ERK1/2 and p38 MAP kinase pathways in CD4+ T cells stimulated through T-cell receptor (TCR) engagement. These pathways are crucial for the transcription of pro-inflammatory genes and the production of cytokines [91]. ERK1/2 and p38 MAPK are part of the MAPK family, which regulates cellular responses to a wide array of stimuli, including stress and cytokines. By inhibiting these pathways, silymarin reduces the production of TNF-α, IL-6, and other pro-inflammatory cytokines, contributing to its overall anti-inflammatory effects [91].

Moreover, silymarin has been found to interfere with the JAK/STAT pathway, another critical signaling mechanism involved in cytokine signaling. The JAK/STAT pathway is activated by various cytokines and growth factors, leading to the transcription of genes involved in cell proliferation, differentiation, and immune responses. By inhibiting this pathway, silymarin further diminishes the inflammatory response, adding another layer of complexity to its anti-inflammatory action [90].

#### 3.2.2. Effects on Macrophages and Other Immune Cells

Silymarin plays a significant role in altering macrophage behavior by encouraging a shift from the pro-inflammatory M1 phenotype to the anti-inflammatory M2 phenotype. This change is important because M1 macrophages are known for producing pro-inflammatory cytokines such as TNF-α, IL-1β, and IL-6, whereas M2 macrophages secrete anti-inflammatory cytokines like IL-4, IL-10, IL-13, and TGF-β. By inducing this switch, silymarin helps to reduce inflammation while supporting tissue repair and maintaining homeostasis [90,92].

The transition from M1 to M2 macrophages involves several key signaling pathways. Silymarin exerts its effects on these pathways by regulating transcription factors and cytokine signaling. For instance, it inhibits the activation of NF-κB and MAPK pathways, which are essential for maintaining the M1 phenotype [92]. By blocking these pathways, silymarin decreases the production of pro-inflammatory cytokines and promotes the expression of genes characteristic of the M2 phenotype, such as arginase-1 and IL-10 [92]. Silymarin also suppresses T-cell proliferation and the production of pro-inflammatory cytokines, including TNF-α, IFN-γ, and IL-12 [90]. This is particularly important in autoimmune and inflammatory conditions, where T-cell activation plays a crucial role. Silymarin’s modulation of T-cell activity involves inhibiting the JAK/STAT and mTOR pathways, which are key for T-cell proliferation and cytokine production. Through this inhibition, silymarin reduces the overall immune response, helping to alleviate inflammation [90].

Furthermore, silymarin affects dendritic cells (DCs), which are important antigen-presenting cells in the immune system. It inhibits the maturation and function of DCs, diminishing their ability to activate T cells. This occurs by downregulating co-stimulatory molecules like CD80 and CD86 and inhibiting the production of IL-12. By impairing the function of DCs, silymarin further contributes to the suppression of the immune response, underscoring its wide-ranging immunomodulatory effects [90].

#### 3.2.3. Inhibition of Inflammasome Activation

Silymarin also inhibits the activation of inflammasomes, particularly the NLRP3 inflammasome, which is crucial for the maturation and release of pro-inflammatory cytokines IL-1β and IL-18. By inhibiting inflammasome activation, silymarin reduces the production of these cytokines, thereby decreasing inflammation [90]. This mechanism is especially important in controlling inflammatory diseases where excessive inflammasome activation is a key pathological feature.

The NLRP3 inflammasome is a multi-protein complex that plays a critical role in the innate immune system. It is triggered by various stimuli, such as microbial infections, cellular damage, and metabolic disruptions. Upon activation, NLRP3 initiates the formation of the inflammasome complex, which includes the adaptor protein ASC and the effector protein caspase-1 [90,93]. Once caspase-1 is activated, it cleaves pro-IL-1β and pro-IL-18 into their active forms, IL-1β and IL-18, which are then released to amplify the inflammatory response [90,93].

Silymarin inhibits the activation of the NLRP3 inflammasome through several mechanisms. It modulates the expression and assembly of the inflammasome components, thus preventing the formation of the active inflammasome complex. This involves the regulation of upstream signaling pathways that are critical for inflammasome activation. For instance, silymarin has been shown to downregulate the TXNIP (thioredoxin-interacting protein) pathway, which is a crucial activator of NLRP3. By reducing TXNIP levels, silymarin prevents the activation of NLRP3 and subsequent inflammasome assembly [90,91].

Additionally, silymarin exerts its effects through the NAD+/SIRT2 pathway. Sirtuins, particularly SIRT2, are known to deacetylate and inactivate key components of the NLRP3 inflammasome. Silymarin enhances the activity of SIRT2, leading to the deacetylation and inhibition of NLRP3, thereby preventing inflammasome activation and reducing IL-1β and IL-18 production [90,91].

Silymarin also impacts mitochondrial function and oxidative stress, both of which are critical for NLRP3 activation. By reducing mitochondrial ROS production and stabilizing mitochondrial integrity, silymarin prevents the mitochondrial dysfunction that typically triggers NLRP3 inflammasome activation. This reduction in oxidative stress is a key aspect of silymarin’s ability to inhibit inflammasome activation [90,91,92].

Silymarin’s inhibition of NLRP3 inflammasome activation results in a significant decrease in the production of IL-1β and IL-18, two key pro-inflammatory cytokines. IL-1β is a critical mediator in the inflammatory process, facilitating the recruitment and activation of immune cells at inflammation sites. IL-18 is essential for inducing IFN-γ production and promoting Th1 responses. By lowering the levels of these cytokines, silymarin effectively reduces the inflammatory response and helps prevent tissue damage [91,93]. Table 2 summarizes the anti-inflammatory activity of berberine and silymarin.

## 4. Health Benefits of Berberine

### 4.1. Health Benefits of Berberine on Cardiovascular Disease Risk Factors

Berberine shows great potential as a supplement in the prevention of cardiovascular disease due to its multiple beneficial effects, including cholesterol reduction, improved endothelial function, blood glucose regulation, and anti-inflammatory and antioxidant properties [94]. Zamani et al. [95] performed a meta-analysis on the effect of berberine supplementation on cardiovascular risk factors in adults and found that berberine supplementation significantly reduced triglyceride levels, total cholesterol, and low-density lipoproteins and increased high-density lipoproteins. The effects of berberine on the glycemic profile included significantly reduced fasting blood glucose, insulin levels, glycated hemoglobin (HbA1c), and insulin resistance index (HOMA-IR). It also reduced systolic blood pressure, weight and body mass index.

These studies underscore berberine’s potential as a therapeutic agent in the prevention and management of cardiovascular diseases, though further clinical trials are needed to fully establish dosage guidelines and long-term safety profiles.

### 4.2. Berberine and Lipid Profile

The liver is integral to the endogenous production of lipids, and berberine serves a regulatory function in hepatic lipid metabolism, which is crucial for the prevention and treatment of metabolic lipid diseases like non-alcoholic fatty liver disease (NAFLD). Berberine’s mechanisms of action involve the reduction of hepatic lipid production, the facilitation of lipid clearance, the enhancement of insulin resistance, the promotion of phosphorylation of AMPK, and the modulation of apoptosis and autophagy in lipid cells, as well as the regulation of epigenetic modifications [96].

CD36, also known as the fat receptor, is a membrane glycoprotein with critical roles in lipid metabolism and the immune response. It is a versatile receptor found on various cell types, including adipocytes, endothelial cells, muscle cells, and macrophages. Additionally, acetyl-CoA carboxylase (ACC), an enzyme vital for fatty acid synthesis, and microsomal triglyceride transfer protein (MTTP) are important regulators of lipid uptake and synthesis in the liver. Berberine reduces circulating lipid levels and liver lipid accumulation by inhibiting these regulators [97,98].

Ju et al. [99] conducted a systematic review and meta-analysis of randomized controlled trials (RCTs) to assess the efficacy and safety of berberine in treating dyslipidemias. The analysis included 16 RCTs with 2147 participants and demonstrated that berberine significantly lowered total cholesterol, low-density lipoprotein cholesterol, and triglyceride levels. It also significantly increased high-density lipoprotein cholesterol, suggesting its potential as an alternative or complementary treatment for hyperlipidemia.

In a similar study, Dong et al. [100] performed a systematic review and meta-analysis of RCTs to examine the impact of berberine on lipid profiles and its safety. The analysis involved 11 RCTs with 874 participants and found that berberine effectively reduced total cholesterol, low-density lipoprotein cholesterol, and triglycerides, while also boosting high-density lipoprotein cholesterol levels. Notably, no serious adverse effects were reported, underscoring berberine’s favorable safety profile.

Numerous studies indicate that berberine can reduce levels of total cholesterol, triglycerides, and low-density lipoprotein cholesterol, suggesting it is a promising option for treating dyslipidemia [101,102].

Berberine’s therapeutic effects can be attributed to several underlying mechanisms of action. Notably, it enhances the expression of LDL receptors within the liver, which aids in the uptake of LDL from the blood, consequently leading to a reduction in LDL cholesterol levels. This mechanism is akin to that of statins, which also promote LDL receptor expression. Furthermore, berberine inhibits the activity of proprotein convertase subtilisin/kexin type 9 (PCSK9), an enzyme that plays a pivotal role in the degradation of LDL receptors. By lowering PCSK9 levels, berberine increases the number of LDL receptors available for the clearance of LDL cholesterol from plasma. This action is comparable to that of FDA- and EMA-approved medications such as evolocumab (Repatha^®^, Amgen, Thousand Oaks, CA, USA) and alirocumab (Praluent^®^, Sanofi, Paris, France), which were both authorized in 2015 [103]. Hepatocytes utilize the hepatic scavenger receptor class B type I (SR-BI) and the low-density lipoprotein receptor (LDLR) to effectively capture and eliminate circulating HDL-C and LDL-C. Additionally, the liver employs the ATP-binding cassette transporter A1 (ABCA1) to facilitate the transfer of cholesterol to apolipoprotein AI (ApoAI), thereby contributing to the formation of HDL-C. The compound berberine influences these lipid metabolic pathways, leading to a reduction in both circulating and hepatic lipid levels [96].

Zhang et al. [104] conducted a meta-analysis to assess the efficacy and safety of berberine in the treatment of hyperlipidemia. The results showed that berberine significantly reduced total cholesterol and low-density lipoprotein cholesterol levels while increasing high-density lipoprotein levels, compared with the placebo group. However, no significant differences were found between the berberine and simvastatin groups regarding low-density and high-density lipoprotein levels. Notably, the combination of berberine and simvastatin was more effective in lowering triglyceride levels than simvastatin alone. Regarding adverse effects, the group receiving berberine, either alone or in combination with simvastatin, experienced fewer side effects such as elevated transaminases and muscle discomfort compared with the control group, though there was a higher incidence of constipation in this treatment group.

Berberine exerts a significant influence on cardiovascular health primarily by modulating lipid metabolism. Numerous studies have demonstrated that berberine effectively lowers levels of LDL cholesterol and triglycerides, while simultaneously facilitating an increase in HDL cholesterol. This lipid-lowering mechanism is largely attributed to the activation of AMPK, an enzyme that plays a pivotal role in maintaining cellular energy balance and regulating lipid and glucose metabolism [105]. The stimulation of AMPK promotes lipid oxidation and enhances glucose uptake, both of which are essential for mitigating cardiovascular risk factors, particularly in individuals suffering from metabolic syndrome or diabetes [104]. Consequently, berberine may contribute to the prevention of atherosclerosis, a leading contributor to cardiovascular disease (CVD) [100].

Wang et al. [106] investigated how berberine supplementation influenced metabolism and lipid storage in black sea bream (*Acanthopagrus schlegelii*) subjected to both high-lipid (HL) and standard diets. The study showed that fish receiving the high-lipid diet supplemented with berberine (HLB) had significantly reduced serum levels of triglycerides (TAG), low-density lipoprotein cholesterol (LDL-C), and alanine aminotransferase activity compared with those on the unsupplemented HL diet. In the HL group, substantial lipid accumulation was observed in the liver, manifesting as enlarged hepatocytes, prominent lipid droplets, and displacement of the nucleus toward the cell’s periphery. The diet supplemented with berberine significantly reduced the expression of hepatic genes involved in lipogenesis, including α-acetyl-CoA carboxylase, sterol regulatory element-binding protein-1, 6-phosphogluconate dehydrogenase, glucose 6-phosphate dehydrogenase, and PPARγ. Simultaneously, genes involved in lipolysis, such as lipoprotein lipase, hormone-sensitive lipase, and carnitine palmitoyltransferase 1a, were upregulated. Interestingly, the pattern of gene expression in muscle tissue showed the opposite trend, with the exception of PPARγ. In summary, berberine supplementation reduced hepatic lipid accumulation by modulating gene expression to decrease fat synthesis and enhance fat breakdown, while it increased muscle lipid content through opposing regulatory mechanisms in muscle tissue compared with the liver.

Another crucial mechanism is berberine’s influence on the gut microbiota. It has been observed that berberine alters the gut microbiota composition, increasing the abundance of beneficial bacteria that produce short-chain fatty acids (SCFAs). These SCFAs can modulate lipid metabolism, further contributing to the reduction in plasma lipid levels [107].

### 4.3. Berberine and Blood Pressure

Berberine’s antihypertensive effects are primarily attributed to its activation of the AMPK pathway, which is essential for energy metabolism and vascular well-being. By activating AMPK, berberine improves endothelial function and facilitates vasodilation, leading to a decrease in systemic vascular resistance, a critical element in the development of hypertension. Moreover, berberine has been found to inhibit the sympathetic nervous system and stimulate the production of nitric oxide, both of which aid in the reduction of blood pressure [108].

Although the evidence derived from human trials is promising, it remains somewhat variable due to differences in study designs, dosing strategies, and the characteristics of patient populations. A systematic review published in 2021 evaluated several RCTs that explored the effects of berberine on blood pressure management. This review included five RCTs and two non-randomized studies, totaling 614 participants. The outcomes suggested that berberine, particularly when paired with other antihypertensive drugs like amlodipine, could result in moderate reductions in blood pressure. However, when assessed against amlodipine alone, berberine did not provide any significant additional benefits [108]. Interestingly, within the same study, a subgroup analysis comparing berberine to metformin, a standard treatment for type 2 diabetes, indicated that berberine led to a statistically significant decline in systolic blood pressure, with a mean reduction of 11.87 mmHg compared with metformin. This suggests that berberine may be particularly beneficial for individuals with both diabetes and hypertension, where insulin resistance and metabolic dysfunction play a critical role in blood pressure regulation.

More recently, a systematic review and meta-analysis published in 2022 [95] further explored the cardiovascular benefits of berberine, with a specific focus on its effects on blood pressure regulation. This analysis, which incorporated data from additional randomized controlled trials, confirmed a significant reduction in systolic blood pressure (WMD = −5.46 mmHg; 95% CI −8.17, −2.76; *p* < 0.001), particularly in individuals with elevated baseline systolic pressure (≥120 mmHg). While the effect on diastolic blood pressure was less pronounced and did not reach statistical significance (WMD = −2.74 mmHg; 95% CI −5.63, 0.15; *p* = 0.063), the findings suggest that berberine may be particularly effective in targeting systolic hypertension. Additionally, the review highlighted that intervention durations longer than eight weeks were associated with more substantial reductions in blood pressure, indicating the importance of sustained supplementation for optimal cardiovascular outcomes. These results build on the prior evidence and suggest that berberine holds promise as a complementary therapy for managing hypertension, particularly in populations with concurrent metabolic disorders such as diabetes, where blood pressure regulation is often compromised.

### 4.4. Berberine and Endothelial Function

Endothelial cells, which form the inner lining of blood vessels, are essential for maintaining vascular health. They are responsible for regulating blood flow, sustaining vessel tone, and inhibiting thrombosis [109]. When endothelial function is compromised, as seen in conditions marked by reduced vasodilation, inflammation, and heightened oxidative stress, it can lead to atherosclerosis and various cardiovascular disorders [109]. Consequently, the significance of endothelial health in assessing cardiovascular risk has prompted increased research into the protective properties of berberine.

The protective role of berberine on the endothelium is largely due to its capacity to influence various essential pathways that govern endothelial function. A significant mechanism involves the activation of AMPK, which is crucial for maintaining cellular energy balance. When berberine activates AMPK, it results in increased production of nitric oxide (NO) through the upregulation of endothelial nitric oxide synthase (eNOS), an enzyme vital for sustaining vascular tone and averting endothelial dysfunction [110,111].

The antioxidant properties of berberine further enhance its protective effects on the endothelium. Oxidative stress, defined as the disproportionate production of ROS in comparison to the body’s antioxidant mechanisms, is a major factor in endothelial damage. Studies have demonstrated that berberine increases the activity of key antioxidant enzymes, such as SOD, and lowers ROS levels, effectively preventing oxidative injury to endothelial cells [112].

Inflammation is a critical driver of endothelial dysfunction, particularly in conditions such as diabetes and metabolic syndrome. Berberine has been shown to exhibit potent anti-inflammatory properties by inhibiting the expression of pro-inflammatory cytokines such as TNF-α and IL-6. Recent studies have demonstrated that berberine inhibits vascular inflammation by downregulating the expression of COX-2 and reducing the levels of CRP, which are key markers of endothelial inflammation [112].

These effects suggest that berberine could serve as a valuable therapeutic option for cardiovascular diseases, particularly in individuals with hypertension and metabolic syndrome.

### 4.5. Berberine as an Antiplatelet Agent

The interest in berberine as a possible agent for inhibiting platelet aggregation has surged in recent years, given its significance in thrombus formation and the prevention of cardiovascular diseases. A variety of reported studies have investigated the impact of berberine and its metabolites on platelet activation, yielding promising findings that suggest its efficacy in preventing thrombosis with minimal associated bleeding risk.

Berberine has been identified as an inhibitor of platelet aggregation through several distinct pathways. A significant mechanism is its ability to block the PI3K/Akt pathway, which is essential for platelet activation. Studies have revealed that berberine can suppress the activation of integrin αIIbβ3, decrease the expression of P-selectin on platelet membranes, and inhibit the binding of fibrinogen to platelets, all of which are integral to the aggregation process. Additionally, both berberine and its major metabolite, berberrubine (M2), have been found to reduce platelet activation without increasing bleeding time, suggesting their potential as safe options for antiplatelet therapy. Moreover, berberine has exhibited notable inhibitory effects on platelet aggregation caused by substances such as ADP, arachidonic acid, and collagen, implying a wide-ranging mechanism of action [113].

Wang et al. [114], using integrated metabolomics and molecular docking, observed that berberine inhibits thrombosis by regulating the catalytic vitamin K cycle.

### 4.6. Effect of Berberine on Type 1 and Type 2 Diabetes Mellitus

Insulin resistance refers to a physiological state in which the cells of the body exhibit diminished sensitivity to insulin, resulting in increased levels of glucose in the bloodstream. If left unaddressed, this condition can progress to type 2 diabetes and various other metabolic disorders. The rising incidence of insulin resistance has sparked significant interest in exploring alternative treatment options, particularly the use of natural substances such as berberine.

Berberine, recognized for its antidiabetic properties, has been shown to lower blood glucose levels, enhance insulin secretion, and diminish both glucose tolerance and insulin resistance through the activation of the AMPK pathway. In addition to its role as an antidiabetic agent, berberine possesses a range of other beneficial activities, including anti-adipogenic, anti-hyperlipidemic, anti-inflammatory, and antioxidant effects. Although numerous studies have explored the effects of berberine, the precise mechanisms underlying its actions remain unclear and warrant further research [115].

Panigrahi et al. [116] investigated the impact of daily oral berberine on glycemic control and insulin resistance in individuals diagnosed with pre-diabetes. After a 12-week intervention involving berberine (HIMABERB^®^), significant reductions in glycemic control markers were observed, with mean fasting plasma glucose (FPG) and 2 h oral glucose tolerance test (2 h-OTGG) levels falling below pre-diabetic thresholds. These findings support the potential of HIMABERB^®^ to delay the progression to diabetes mellitus.

One of the well-established mechanisms by which berberine lowers blood glucose levels is through the activation of the AMPK signaling pathway. More recently, berberine has been shown to function as an insulin secretagogue by directly binding to KCNH6 potassium channels. This interaction leads to prolonged glucose-dependent depolarization of the cell membrane, which in turn stimulates insulin secretion [51,117]. In a randomized, double-blind, placebo-controlled clinical trial conducted by Pérez-Rubio et al. [118], the effects of berberine on glucose regulation in metabolic syndrome were evaluated. The study demonstrated that berberine administration significantly reduced the area under the curve (AUC) for glucose, indicating improved glycemic control. Additionally, insulin secretion decreased while insulin sensitivity improved, as reflected by enhancements in the insulinogenic and Matsuda indices. These findings highlight berberine’s potential in improving glucose metabolism, particularly in individuals with metabolic syndrome.

In summary, berberine demonstrates significant potential as a natural compound for enhancing insulin sensitivity and addressing insulin resistance. Its mechanism of action, which includes the activation of AMPK, modulation of glucose metabolism, and reduction of inflammation, positions it as a valuable candidate for tackling the fundamental causes of insulin resistance. Clinical studies have substantiated its effectiveness in lowering insulin levels and improving HOMA-IR scores, while its safety profile is largely positive.

### 4.7. Effect of Berberine on Obesity

A primary mechanism through which berberine addresses obesity involves the regulation of glucose metabolism and the enhancement of insulin sensitivity. By stimulating the AMPK pathway, berberine facilitates the uptake of glucose by cells, thereby diminishing insulin resistance. This process not only curtails excessive appetite but also leads to a reduction in caloric consumption, thereby supporting weight-loss efforts. Furthermore, by downregulating critical transcription factors such as PPARγ and SREBP1, berberine inhibits the differentiation of adipocytes, which involves the transformation of pre-adipocytes into mature fat-storing cells. This inhibition results in a decrease in the generation of new adipose tissue and promotes the oxidation of fat, ultimately contributing to a reduction in overall body fat [119].

Recent studies have underscored the capacity of berberine to modify the gut microbiota composition, a factor that significantly influences obesity. Berberine enhances the diversity of gut microbes, fostering the proliferation of beneficial bacteria that support weight management. Administered at a dosage of 500 mg per day, berberine was demonstrated to affect microbial communities, thereby enhancing metabolic health and facilitating weight loss [120].

To conclude, berberine presents a noteworthy potential as a natural intervention for obesity, primarily by modulating glucose and lipid metabolism, preventing adipocyte development, and altering gut microbiota composition. While the outcomes from animal research and initial human studies are optimistic, it is imperative to conduct further extensive clinical trials to ascertain its long-term safety and effectiveness. When used alongside a nutritious diet and active lifestyle, berberine could prove to be a significant asset in the management of obesity.

### 4.8. The Effect of Berberine on Apoptosis

Apoptosis, or the process of programmed cell death, is a highly controlled biological phenomenon that allows organisms to preserve cellular homeostasis while discarding damaged or unnecessary cells. The dysregulation of this process is associated with a variety of diseases, including cancer, neurodegenerative disorders, cardiovascular diseases, and autoimmune conditions. In recent years, there has been growing interest in berberine for its potential to influence the mechanisms of apoptosis.

Berberine influences apoptosis through a variety of mechanisms, functioning both as an inducer and inhibitor depending on the specific cellular environment. A key pathway through which berberine promotes apoptosis is the mitochondrial or intrinsic pathway. Research indicates that berberine increases the permeability of the mitochondrial membrane, leading to the release of cytochrome c into the cytosol. This release initiates the activation of caspase-9, which subsequently activates downstream effector caspases, including caspase-3, that carry out the apoptotic process by cleaving multiple cellular substrates [121].

Furthermore, berberine has the capacity to influence apoptosis by modulating the expression of Bcl-2 family proteins, which play a crucial role in regulating mitochondrial membrane permeability. Research has shown that berberine decreases the levels of anti-apoptotic proteins, including Bcl-2 and Bcl-xL, while simultaneously increasing the expression of pro-apoptotic proteins such as Bax and Bak [122].

Berberine plays a significant role in apoptosis through the extrinsic pathway, which is associated with death receptor signaling. This pathway is triggered by the interaction of death ligands, such as FasL or TNF-α, with their respective receptors on the cell surface, resulting in the formation of the death-inducing signaling complex (DISC). Evidence suggests that berberine promotes the expression of death receptors like Fas and TRAIL-R, thereby increasing the sensitivity of cancer cells to apoptosis induced by immune cells or therapeutic strategies [123].

Recent research in the field of cardiovascular diseases indicates that berberine may serve a dual purpose; it not only safeguards against apoptosis but also facilitates the regeneration and repair of injured cardiac tissue. For example, some studies have shown that berberine enhances the survival and proliferation of endothelial progenitor cells (EPCs), which are essential for vascular repair and regeneration after ischemic events. By influencing apoptotic pathways and bolstering regenerative mechanisms, berberine presents a promising therapeutic option for individuals with cardiovascular conditions, especially those in the recovery phase following a myocardial infarction [124,125].

Recent investigations have started to examine the potential of berberine to affect apoptosis via epigenetic mechanisms, which entail modifications in gene expression that do not involve changes to the DNA sequence itself. The regulation of epigenetic factors is essential in the context of neurodegenerative diseases. Research indicates that berberine can influence the expression of microRNAs (miRNAs), which are small non-coding RNA molecules that play a significant role in the post-transcriptional regulation of gene expression. Notably, berberine has been observed to enhance the expression of neuroprotective miRNAs that specifically target pro-apoptotic genes, thereby reducing apoptosis in neural cells [126].

While berberine is known for its pro-apoptotic effects in cancer, it paradoxically shows anti-apoptotic characteristics in neurodegenerative conditions. Studies have demonstrated that in models of Alzheimer’s disease, berberine protects neurons from undergoing apoptosis by alleviating oxidative stress, curtailing inflammation, and influencing mitochondrial functions [127]. This neuroprotective action is crucial, especially considering the role that apoptosis plays in the development of neurodegenerative diseases, where increased neuronal death is associated with cognitive decline. Additionally, berberine’s protective effects extend to diabetic neuropathy and neuropathic pain, where it has been shown to reduce neural apoptosis and alleviate symptoms [128]. Berberine reduces nerve damage through its antioxidant and anti-inflammatory effects by decreasing ROS production, protecting mitochondrial function, and modulating pro-inflammatory cytokines like TNF-α and IL-1β [24]. Additionally, berberine improves nerve conduction and reduces pain severity. Its modulation of ion channels and neuropeptides, such as TRPV1 (transient receptor potential vanilloid 1) receptors, further contributes to alleviating neuropathic pain, making it a valuable therapeutic option for managing diabetic neuropathy [129]. Table 3 summarizes the health benefits of berberine.

## 5. Health Benefits of Silymarin

Since inflammation increases oxidative stress in most pathologies, reducing inflammation and oxidation levels by taking silymarin could help to control various pathologies.

### 5.1. Silymarin and Hepatic Diseases

Silymarin has been used since ancient times in the treatment of hepatotoxicity and liver diseases. Nowadays, it is one of the most widely used active ingredients as a supplement in this type of pathology (steatosis and alcohol-related liver disease as well as food poisoning caused by fungus) [61].

While the precise mechanisms underlying silymarin’s actions remain to be fully clarified, research indicates that multiple pathways are likely to be involved. Its antioxidant properties are particularly notable, as silymarin neutralizes free radicals and affects enzyme systems related to glutathione and superoxide dismutase, primarily through the increased expression and activation of the nuclear transcription factor erythroid 2-related factor 2 (Nrf2). Additionally, it exhibits anti-inflammatory effects by inhibiting the production of leukotrienes and prostaglandins. Silymarin also has antifibrogenic properties, as it reduces collagen synthesis and helps prevent liver fibrosis. Furthermore, it promotes cell regeneration by enhancing protein synthesis and supporting hepatocyte regeneration [131,132,133,134].

Both oxidative stress and inflammation are key factors in the progression of chronic liver diseases such as alcoholic liver disease (ALD), NAFLD, and drug-induced liver injury. Silymarin may help to improve both aspects. Numerous studies corroborate this [135,136,137]. In 2017, Avelar et al. found in a systematic review with meta-analysis that silymarin significantly but not clinically relevantly reduced liver enzymes (ALT, AST, GGT) [138]. The anti-inflammatory and antioxidant effects of silymarin flavonoids may underlie the improvement in non-alcoholic fatty liver disease [139].

Silymarin has been extensively demonstrated to protect against liver damage caused by alcohol, toxins, and various drugs. Its protective mechanism includes neutralizing free radicals and regulating enzymes involved in cellular damage, fibrosis, and cirrhosis. These hepatoprotective effects have been documented in clinical studies including patients suffering from both alcoholic and non-alcoholic fatty liver disease, as well as those with cirrhosis [135,140].

### 5.2. Silymarin and Diabetes

Silymarin has also demonstrated effectiveness in managing diabetes. Studies have shown its ability to regulate blood glucose levels and reduce fasting blood sugar, hemoglobin A1C, insulin, low-density lipoprotein cholesterol, and malondialdehyde levels, while increasing high-density lipoprotein cholesterol levels [139,141].

DM is a widespread epidemic in Western societies, which has led to an increase in research on the antidiabetic properties of silymarin. Recent studies have focused on its effects in type 2 diabetes mellitus (T2DM), exploring its potential to improve glycemic control, lipid profiles, and reduce oxidative stress.

Regarding improvement in glycemic control, silymarin supplementation significantly reduced fasting blood glucose (FBG) and HbA1c levels in T2DM patients [141,142]. Also, silymarin improves insulin sensitivity and reduces insulin resistance, as indicated by lower HOMA-IR scores [142,143].

At level of the lipid profile modulation, silymarin has been shown to decrease LDL cholesterol and triglyceride levels while increasing HDL cholesterol in T2DM patients [141,142]. However, some studies report no significant effect on total cholesterol and triglyceride concentrations [144].

It has been shown that silymarin can decrease oxidative stress, thus reducing ROS formation, by decreasing the ROS-producing enzymes. Sylimarin increases SOD activity and protects from cell injury, upregulating the mitochondrial membrane potential [145]. Also, silymarin enhances antioxidant indices such as SOD, GPx, and total antioxidant capacity (TAC). It also reduces markers of oxidative stress, including malondialdehyde (MDA) and high-sensitivity CRP (hs-CRP) [142,146].

### 5.3. Silymarin in Cardiovascular Diseases

Another potential benefit of silymarin consumption is in relation to CVDs. Several studies have shown that silymarin intake may improve cardiovascular risk factors, due to its improved lipid profile, antioxidant activity, and anti-inflammatory activity as well as its positive effect on endothelial dysfunction.

Soleymani et al., in a systematic review, indicate that silymarin significantly improves lipid profiles by reducing total cholesterol, triglycerides, and low-density lipoprotein cholesterol while increasing high-density lipoprotein cholesterol [147]. Kadoglou et al. [148], in another review, concluded that silymarin also helps in lowering fasting blood glucose and hemoglobin A1C levels, which are beneficial for patients with type 2 diabetes mellitus, a major risk factor for CVDs.

The importance of oxidative stress in cardiovascular disease should not be overlooked. Silymarin’s antioxidant properties help to improve these pathologies, protecting against oxidative stress-induced cardiovascular conditions such as hypertension, atherosclerosis, and cardiac toxicity. It has been shown to reduce oxidative stress markers and improve histopathological changes in heart tissues in diabetic and cirrhotic rat models [146,149].

Silymarin’s anti-inflammatory properties are also beneficial for cardiovascular diseases. Several studies have explored this connection, including research by Al-Rasheed et al., which found that silymarin improved levels of inflammatory and immunological biomarkers such as TNF-α, IFNγ, IL-6, and CRP. This highlights the anti-inflammatory potential of silymarin in protecting against myocardial injury [150].

**Figure 2:**
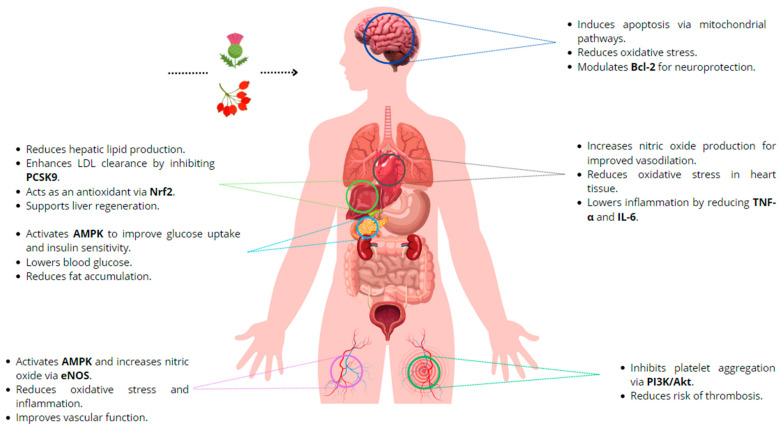
Systemic health effects of berberine and silymarin.

Silymarin has been shown to reduce asymmetric dimethylarginine (ADMA) in mice at the level of endothelial dysfunction. This reduction means that nitric oxide reductase inhibition is reduced, and nitric oxide formation is therefore not affected [145]. In addition, Palomino et al. [151] reported a protective effect of silymarin on endothelial cells exposed to high glucose concentrations, which may be relevant for preventing diabetes-induced cardiovascular damage. Their study indicated that silymarin restored glutathione levels and modulated antioxidant enzyme activities, offering protection against oxidative stress in endothelial cells. Table 4 summarizes the health benefits of silymarin, and Figure 2 summarizes the health benefits of both berberine and silymarin.

## 6. Clinical Trials Evaluating the Effects of Berberine and Silymarin: Synergistic and Individual Outcomes

### 6.1. Synergistic Effects of Berberine and Silymarin

The evidence of the synergistic effect of berberine and silymarin is emerging; however, their effects as separated compounds have been widely proven. The effectiveness of the combination of berberine with silymarin was analyzed in 136 obese subjects with type 2 diabetes and metabolic syndrome. After 6 months of treatment, both compounds were associated with significantly improved fasting blood glucose and insulin, HOMA-IR, total cholesterol, HDL and LDL cholesterol, triglycerides, uric acid, body mass index (BMI), waist circumference, waist to hip ratio, and % of abdominal fat assessed via bioelectrical impedance significantly improved compared with the placebo [152].

In another study, 137 euglycemic, dyslipidemic subjects with previous adverse events to statins at high doses were randomized to receive placebo or *B. aristata*/*S. marianum* 588/105 mg additional to their half-statin dose for six months. The combination proved to be effective and safe for those patients that did not tolerate statins’ side effects [153,154].

Additionally, in polygenic hypercholesterolemia, 2 month treatment with combined berberine and sylimarin was compared with atorvastatin 10 mg administrated in a matched control group. The complex reduced LDL-C levels with an effect like that induced by 10 mg of statins and ex vivo improved the functional profile of lipoproteins with antiatherogenic action [155].

### 6.2. Effects of Berberine or Silymarin as Single Agents

A randomized, double-blinded, placebo-controlled trial was conducted for 12 weeks among 34 individuals with prediabetes as defined by the American Diabetes Association. Berberine, HIMABERB^®^ 500 mg, or placebo were given three times daily to the participants. Markers of glycemic control and physical parameters were assessed in both groups on days 0, 28, 56, and 84. The berberine group showed significant reductions in all glycemic control markers at various time points throughout the study and at the endpoint, compared with both baseline levels and the control group. The treatment was well tolerated and safe for all participants [116]. In another study including 40 patients with type 2 diabetes, silymarin supplementation significantly improved antioxidant outcomes compared with the placebo, observed through an increment in superoxide dismutase, glutathione peroxidase activity, and total antioxidant capacity [156].

In a study of 49 overweight patients (28 females and 21 males), participants were randomly assigned to either the berberine group (n = 24) or placebo (n = 25). At baseline, +30 days, and +60 days, glycemia was evaluated as the primary endpoint, with lipidic and other glycemic biochemistry parameters such as LDL/HDL or glycated hemoglobin, to name a few, as secondary endpoints. After 60 days of treatment, significant differences between the active treatment and placebo groups were observed in several parameters, including glycemia, total cholesterol, total cholesterol/HDL ratio, triglycerides, insulin, ApoB/ApoA ratio, visceral adipose tissue (VAT), and fat mass [157].

Moreover, a clinical trial involving patients with moderate hypercholesterolemia and cardiovascular risk treated for 3 months with Lipok^®^—a complex formulation containing 3.75 mg of monacolin K, 515 mg of berberine, and 50 mg of coenzyme Q10 per tablet—or placebo, showed reductions in LDL-C, non-HDL-C, and total cholesterol with the active treatment [154].

An open-label, parallel, randomized controlled trial involving 300 patients with H. pylori infection assigned participants to receive one of three treatments: berberine triple therapy (berberine 500 mg, amoxicillin 1000 mg, vonoprazan 20 mg), vonoprazan quadruple therapy (vonoprazan 20 mg, amoxicillin 1000 mg, clarithromycin 500 mg, colloidal bismuth tartrate 220 mg), or standard quadruple therapy (rabeprazole 10 mg, amoxicillin 1000 mg, clarithromycin 500 mg, colloidal bismuth tartrate 220 mg), all administered twice daily for 14 days. The per-protocol (PP) analysis revealed H. pylori eradication rates of 81.4% (70/86) for berberine, 86.5% (77/89) for vonoprazan, and 78.4% (69/88) for rabeprazole, indicating that berberine was as effective as the other two therapies evaluated [158]. Another randomized controlled open-label trial of 524 patients assigned to either berberine triple therapy or standard quadruple therapy for 14 days showed similar eradication rates in both intention-to-treat and per-protocol analyses (both *p* > 0.636), demonstrating the noninferiority of berberine treatment [159].

In the clinical trial by Pérez Rubio et al. involving 24 patients with metabolic syndrome, berberine administration (500 mg three times daily for 3 months) significantly improved BMI, waist circumference, and systolic blood pressure, with a 36% remission rate of metabolic syndrome in the treatment group [118].

Silymarin has also been studied independently. In a study involving 59 participants with a BMI of ≤34.9 kg/m^2^, subjects were randomized to receive supplements with or without silymarin. The group receiving silymarin showed a reduction in waist circumference both at the middle abdomen and iliac crest [160].

### 6.3. Effects in Immunocompromised Patients and Hepatoprotection

The synergistic effects of both components still remain in this field; however, it is worth discussing some of the uses of each compound consistently described in the literature. In a double-blind, placebo-controlled trial, 36 adult participants with metabolic syndrome and viral suppressed HIV took daily either berberine 500 mg or placebo for 5 months. The main outcomes evaluated weight reduction, insulin resistance decrease, and lipid profile improvement. The treatment group showed a reduction in weight and body mass index, and lower insulin resistance [161]. Another disease where berberine has been used is primary sclerosing cholangitis (PSC), a fibroinflammatory disease of the bile ducts leading to cirrhosis and hepatic decompensation that has few medications approved by drug regulatory agencies. After 18-week proof-of-concept study, only those treated with 500 mg of berberine experienced alkaline phosphatase reductions that remained stable until the end of study [162].

Also, the immune and inflammatory effects of berberine were assessed in 59 schizophrenia patients, who were randomized to receive placebo or berberine (900 mg/day) for 2 months; berberine improved the negative symptom scores and decreased the plasma CRP concentration [163]. Furthermore, 132 patients with schizophrenia or bipolar disorder who underwent long-term treatment with olanzapine and whose intestinal flora was affected or who presented mild metabolic disorders received berberine or placebo tablets for 12 weeks. Those that received the active component had improved intestinal flora and presented lower metabolic side effects [164].

Those results were confirmed by a subsequent work, where the authors reported that in a sample of 113 patients with schizophrenia, those taking berberine for 3 months presented lesser weight gain or metabolic syndrome symptoms [165].

A randomized clinical trial in 68 patients with Parkinson’s disease explored the effect of berberine hydrochloride on the diversity of intestinal. After continuous treatment for 3 months, patients in the treatment group had better intestinal flora and lower levels inflammatory markers such as IL-8, IL-6, and TNF-α [166].

In a cross-over study, 82 β-thalassemia major patients took silymarin (140 mg, 3 times a day) or placebo for 12 weeks. After a 2-week washout period, they were crossed over to the other group. The combination of iron chelation therapy with silymarin can improve inflammatory status in patients, decreasing IL-6 and CRP and increasing IL-10 [167].

Furthermore, the main effect of silymarin as a solo treatment is its capacity for reducing hepatotoxicity in cirrhotic patients [168], breast cancer patients treated with doxorubicin [169], or hepatitis C patients [170]. In all these reports, a trend to improve hepatic markers is described, but without a statistical significance. Its hepatoprotective effects were proven in a clinical trial in people with multiple sclerosis, where 48 participants were allocated randomly to either the placebo or silymarin arm for six months. Those in the active component group had decreased levels of ALT and AST, malondialdehyde contents, and also an increment in total antioxidant capacity and increased total thiol groups in the serum [156]. Table 5 summarizes clinical trials showing the synergistic effect of berberine and silymarin.

## 7. Conclusions and Future Research Lines

Berberine and silymarin are recognized for their substantial antioxidant and therapeutic potential. Berberine enhances endogenous antioxidant defenses by increasing the activity of enzymes such as SOD, CAT, and GPx, along with activating the AMPK and Nrf2 pathways, contributing to reducing oxidative stress and protecting cells from damage. Additionally, its free radical scavenging activity and ability to chelate metal ions further support its antioxidant properties. Similarly, silymarin exerts significant antioxidant effects by inhibiting ROS-producing enzymes, stabilizing mitochondrial membranes, and activating the Nrf2 pathway, improving cellular redox balance through the enhancement of antioxidant enzymes and modulation of stress response genes.

The combination of berberine and silymarin holds potential for addressing metabolic and inflammatory diseases, particularly in conditions like diabetes, cardiovascular disorders, and neurodegenerative diseases, where oxidative stress plays a critical role. However, the poor bioavailability of both berberine and silymarin remains a significant challenge. Despite their potent in vitro antioxidant activities, their clinical efficacy is limited by low absorption and rapid metabolism.

Recent advancements in pharmaceutical formulations, such as nanoparticle-based delivery systems, phytosomes, and liposomal formulations, have been developed to enhance the bioavailability of these compounds. These modern formulations improve the solubility and absorption of berberine and silymarin, potentially increasing their therapeutic effects in vivo. Future research should continue to explore and optimize these delivery technologies to maximize the efficacy of berberine and silymarin in clinical applications.

Furthermore, future studies are required to determine the optimal doses and treatment regimens that can maximize their antioxidant and anti-inflammatory effects in different pathologies. Clinical trials exploring the combination of berberine and silymarin in metabolic and cardiovascular diseases could provide important insights into their use within integrated therapeutic strategies.

## Figures and Tables

**Table 1:** Summary of Antioxidant Activity of Berberine and Silymarin.

Extract	Function	Mechanism	References
Berberine	Antioxidant	Enhancement of endogenous antioxidant enzymes (SOD, CAT, GPx) via Nrf2 and AMPK pathways. Upregulation of antioxidant gene expression and enzyme stabilization.	[20,22,23,24,27,29,30]
		Direct scavenging of free radicals by donating electrons or hydrogen atoms, reducing oxidative stress and preventing lipid peroxidation, protein oxidation, and DNA strand breaks.	[14,23,32]
		Chelation of transition metal ions (iron, copper), preventing hydroxyl radical formation via Fenton reactions, thereby protecting cellular structures and maintaining redox balance.	[22,41]
		Activation of Nrf2 leading to enhanced expression of antioxidant genes, including SOD, CAT, GPx, and HO-1, reducing oxidative stress and inflammation.	[43,45]
		AMPK activation reduces NADPH oxidase activity and mitochondrial ROS production and upregulates antioxidant enzymes, protecting cells from oxidative damage and improving energy homeostasis.	[51,52,59]
Sylimarine		Direct scavenging of free radicals (HOCl, hydroxyl radicals), reducing oxidative damage and inflammation. Inhibition of ROS-producing enzymes like NADPH oxidase and xanthine oxidase.	[61,62,63,64,65,66]
		Stabilization of mitochondrial membranes, optimizing electron transport chain efficiency, reducing electron leakage, and maintaining ATP production, thus lowering oxidative stress.	[62,67,68]
		Activation of Nrf2 and NF-κB pathways, upregulating antioxidant enzymes (SOD, CAT, GPx, HO-1) and reducing inflammation through modulation of pro-inflammatory cytokines.	[62,72]
		Activation of vitagenes (HSPs, Trx, sirtuins), ensuring cellular defense against oxidative stress, improving protein folding, DNA repair, and energy metabolism, contributing to cellular longevity.	[76]

AMPK: adenosine monophosphate-activated protein kinase, ATP: adenosine triphosphate, CAT: catalase, DNA: deoxyribonucleic acid, GPx: glutathione peroxidase, HO-1: heme oxygenase-1, HOCl: hypochlorous acid, HSPs: heat shock proteins, NADPH: nicotinamide adenine dinucleotide phosphate, NF-κB: nuclear factor kappa-light-chain-enhancer of activated B cells, Nrf2: nuclear factor erythroid 2-related factor 2, ROS: reactive oxygen species, SOD: superoxide dismutase, Trx: thioredoxin.

**Table 2:** Summary of Anti-inflammatory Activity of Berberine and Silymarin.

Extract	Function	Mechanism	References
Berberine	Anti-inflammatory	Inhibition of NF-κB and AP-1 pathways, reducing the production of pro-inflammatory cytokines (TNF-α, IL-1β, IL-6, MCP-1, COX-2, iNOS).	[78,79]
		Activation of AMPK, which downregulates pro-inflammatory genes and inhibits the mTOR pathway, further reducing cytokine expression.	[51,52,79,80]
		Modulation of gut microbiota, promoting Treg differentiation and inhibiting Th17 cell differentiation, reducing inflammation.	[83,85,86]
		Inhibition of MAPK pathways (ERK, JNK, p38 MAPK), decreasing the production of pro-inflammatory cytokines (TNF-α, IL-6, IL-1β) and ROS.	[79,82]
		Shift from pro-inflammatory M1 macrophages to anti-inflammatory M2 phenotype, reducing cytokine production and promoting tissue repair.	[24,81,83]
Sylimarine		Modulation of cytokine signaling pathways, inhibiting NF-κB and MAPK pathways, reducing pro-inflammatory mediators (TNF-α, IL-1β, IL-6) and nitric oxide production.	[90,91]
		Inhibition of TLR4/NF-κB-mediated signaling, decreasing expression of inflammatory cytokines and chemokines (IL-12, IL-23, CCL4, CXCL10).	[90]
		Shift from pro-inflammatory M1 to anti-inflammatory M2 macrophages, promoting IL-4, IL-10, and TGF-β production and reducing inflammation.	[90,92]
		Inhibition of NLRP3 inflammasome activation, reducing production of IL-1β and IL-18, key mediators in inflammation and autoimmunity.	[90,93]
		Impacts on immune cells, inhibiting T-cell proliferation, dendritic cell maturation, and cytokine production, thus suppressing immune responses.	[90,92]

AMPK: adenosine monophosphate-activated protein kinase, AP-1: activator protein 1, CCL4: chemokine (C-C motif) ligand 4, COX-2: cyclooxygenase-2, CXCL10: C-X-C motif chemokine ligand 10, ERK: extracellular signal-regulated kinase, IL-1β: interleukin-1 beta, IL-4: interleukin-4, IL-6: interleukin-6, IL-10: interleukin-10, IL-12: interleukin-12, IL-18: interleukin-18, IL-23: interleukin-23, iNOS: inducible nitric oxide synthase, JNK: c-Jun N-terminal kinase, MAPK: mitogen-activated protein kinase, MCP-1: monocyte chemoattractant protein-1, mTOR: mammalian target of rapamycin, NF-κB: nuclear factor kappa-light-chain-enhancer of activated B cells, NLRP3: Nod-like receptor family pyrin domain containing 3, ROS: reactive oxygen species, Th17: T-helper 17 cells, TLR4: toll-like receptor 4, TNF-α: tumor necrosis factor-alpha, Treg: regulatory T cells, TGF-β: transforming growth factor-beta.

**Table 3:** Summary of Health Benefits of Berberine.

Health Aspect	Mechanism	References
Cardiovascular disease risk factors	Reduction in triglycerides, total cholesterol, and LDL; increase in HDL; regulation of blood glucose and insulin; anti-inflammatory and antioxidant properties.	[94]
Lipid profile	Decrease in hepatic lipid production and circulating lipid levels; enhancement of LDL receptor expression; inhibition of PCSK9, improving lipid clearance.	[95,96,97,98,99,100,101,102]
Blood pressure	Activation of AMPK pathway, improving endothelial function and vasodilation; inhibition of sympathetic nervous system; stimulation of nitric oxide production.	[107]
Endothelial function	Activation of AMPK and increased nitric oxide production (via eNOS); reduction of oxidative stress and inflammation, improving vascular health.	[110,111]
Antiplatelet agent	Inhibition of platelet aggregation via PI3K/Akt pathway; suppression of integrin αIIbβ3 activation, reducing the risk of thrombosis without increasing bleeding risk.	[112,113]
Type 1 and type 2 diabetes mellitus	Activation of AMPK pathway, improving glucose uptake and insulin sensitivity; reduction in blood glucose levels; improvement in insulin resistance.	[114,115,116,117]
Obesity	Regulation of glucose metabolism; improvement in insulin sensitivity; inhibition of adipocyte differentiation and modulation of gut microbiota composition, leading to reduced fat accumulation and weight loss.	[118,119]
Apoptosis	Induction of apoptosis via mitochondrial (intrinsic) and death receptor (extrinsic) pathways; modulation of Bcl-2 family proteins; potential protective effects in neurodegenerative conditions through anti-apoptotic actions.	[120,121,122,123,124,125,126,130]

AMPK: adenosine monophosphate-activated protein kinase, Bcl-2: B-cell lymphoma 2, eNOS: endothelial nitric oxide synthase, HDL: high-density lipoprotein, LDL: low-density lipoprotein, PCSK9: proprotein convertase subtilisin/kexin type 9, PI3K: phosphoinositide 3-kinase, Akt: protein kinase B.

**Table 4:** Summary of Health Benefits of Silymarin.

Health Aspect	Mechanism	References
Hepatic diseases	Antioxidant action (neutralizes free radicals via Nrf2 activation); anti-inflammatory (inhibition of leukotrienes and prostaglandins); antifibrotic (reduces collagen production); promotes hepatocyte regeneration.	[61,127,131,132,133,134,135,136,137,138,139]
Diabetes	Improves glycemic control (reduces fasting blood glucose, HbA1c, insulin levels); modulates lipid profile (decreases LDL, triglycerides, increases HDL); antioxidant effects (increased SOD, GPx, TAC, reduced MDA and hs-CRP).	[138,140,141,142,143,144,145]
Cardiovascular diseases	Improves lipid profile (reduces total cholesterol, triglycerides, LDL; increases HDL); antioxidant properties (reduces oxidative stress markers); anti-inflammatory effects (reduces TNF-α, IL-6, CRP); protects against endothelial dysfunction.	[142,146,147,148,149]

CRP: C-reactive protein, GPx: glutathione peroxidase, HbA1c: hemoglobin A1c, HDL: high-density lipoprotein, hs-CRP: high-sensitivity C-reactive protein, IL-6: interleukin-6, LDL: low-density lipoprotein, MDA: malondialdehyde, Nrf2: nuclear factor erythroid 2-related factor 2, SOD: superoxide dismutase, TAC: total antioxidant capacity, TNF-α: tumor necrosis factor-alpha.

**Table 5:** Clinical Trials Showing Synergistic Effect of Berberine and Silymarin.

Condition/Study Group	Main Findings	References
Type 2 diabetes mellitus and obesity	Combination of berberine and silymarin in 136 obese subjects with type 2 diabetes significantly improved fasting blood glucose, insulin, HOMA-IR, lipid profile, BMI, waist circumference, and % of abdominal fat compared with the placebo.	[152]
Dyslipidemia and statin intolerance	Combination of berberine/silymarin with low-dose statins for six months significantly reduced LDL-C levels in patients with previous adverse reactions to high-dose statins, showing effectiveness and safety.	[153,154]
Polygenic hypercholesterolemia	Combination of berberine and silymarin reduced LDL-C levels with effects comparable to 10 mg of atorvastatin, and improved lipoprotein function with antiatherogenic action in 53 patients.	[155]

BMI: body mass index, HOMA-IR: homeostatic model assessment of insulin resistance, LDL-C: low-density lipoprotein cholesterol.
